# Provision of medical supply kits to improve quality of antenatal care in Mozambique: a stepped-wedge cluster randomised trial

**DOI:** 10.1016/S2214-109X(17)30421-7

**Published:** 2017-12-12

**Authors:** Ana Pilar Betrán, Eduardo Bergel, Sally Griffin, Armando Melo, My Huong Nguyen, Alicia Carbonell, Santos Mondlane, Mario Merialdi, Marleen Temmerman, A Metin Gülmezoglu, WHO Antenatal Care Trial in Mozambique Research Group, Alicia Aleman, Fernando Althabe, Adriano Biza, Beatrice Crahay, Leonardo Chavane, Mercedes Colomar, Therese Delvaux, Ussumane Dique Ali, Lucio Fersurela, Diederike Geelhoed, Ingeborg Jille-Taas, Celsa Regina Malapende, Célio Langa, Nafissa Bique Osman, Jennifer Requejo, Geraldo Timbe

**Affiliations:** a UNDP/UNFPA/UNICEF/WHO/World Bank Special Programme of Research, Development and Research Training in Human Reproduction, Department of Reproductive Health and Research, World Health Organization, Geneva, Switzerland; b Institute for Clinical Effectiveness and Health Policy, Buenos Aires, Argentina; c International Center for Reproductive Health, Maputo, Mozambique; d Mozambique Ministry of Health, Maputo, Mozambique; e Mozambique Country Office, World Health Organization, Maputo, Mozambique; f Consultório de Estatística e Serviço de Soluções, Maputo, Mozambique; g BD Global Health, Franklin Lakes, NJ, USA; h International Center for Reproductive Health Global, Ghent, Belgium; i Aga Khan University East Africa, Nairobi, Kenya

## Abstract

**Background:**

High levels of maternal and newborn mortality and morbidity remain a daunting reality in many low-income countries. Several interventions delivered during antenatal care have been shown to improve maternal and newborn outcomes, but stockouts of medical supplies at point of care can prevent implementation of these services. We aimed to evaluate whether a supply chain strategy based on the provision of kits could improve quality of care.

**Methods:**

We did a pragmatic, stepped-wedge, cluster-randomised controlled trial at ten antenatal care clinics in Mozambique. Clinics were eligible if they were not already implementing the proposed antenatal care package; they served at least 200 new pregnant women per year; they had Maternal and Child Health (MCH) nurses; and they were willing to participate. All women attending antenatal care visits at the participating clinics were included in the trial. Participating clinics were randomly assigned to shift from control to intervention on prespecified start dates. The intervention involved four components (kits with medical supplies, a cupboard to store these supplies, a tracking sheet to monitor stocks, and a one-day training session). The primary outcomes were the proportion of women screened for anaemia and proteinuria, and the proportion of women who received mebendazole in the first antenatal care visit. The intervention was delivered under routine care conditions, and analyses were done according to the intention-to-treat principle. This trial is registered with the Pan African Clinical Trial Registry, number PACTR201306000550192.

**Findings:**

Between March, 2014, and January, 2016, 218 277 antenatal care visits were registered, with 68 598 first and 149 679 follow-up visits. We found significant improvements in all three primary outcomes. In first visits, 5519 (14·6%) of 37 826 women were screened for anaemia in the control period, compared with 30 057 (97·7%) of 30 772 in the intervention period (adjusted odds ratio 832·40; 99% CI 666·81–1039·11; p<0·0001); 3739 (9·9%) of 37 826 women were screened for proteinuria in the control period, compared with 29 874 (97·1%) of 30 772 in the intervention period (1875·18; 1447·56–2429·11; p<0·0001); and 17 926 (51·4%) of 34 842 received mebendazole in the control period, compared with 24 960 (88·2%) of 28 294 in the intervention period (1·88; 1·70–2·09; p<0·0001). The effect was immediate and sustained over time, with negligible heterogeneity between sites.

**Interpretation:**

A supply chain strategy that resolves stockouts at point of care can result in a vast improvement in quality during antenatal care visits, when compared with the routine national process for procurement and distribution of supplies.

**Funding:**

Government of Flanders and the UNDP/UNFPA/UNICEF/WHO/World Bank Special Programme of Research, Development and Research Training in Human Reproduction.

## Introduction

Although progress in reduction of maternal mortality in the past two decades has been systematically documented, it remains a daunting challenge, particularly in low-resource countries.^1^ If effectively implemented, antenatal care can be an important contributor to the reduction of maternal mortality.^2^, ^3^, ^4^ Additionally, in many poor settings, antenatal care constitutes one of the few times women might seek contact with the health system during their reproductive life.^4^, ^5^ For this reason, antenatal care represents a unique opportunity to inform, educate, and reach women with a number of interventions that can be vital for maternal and perinatal health.

In low-resource settings, multiple factors hinder the delivery of evidence-based practices for antenatal care.^6^, ^7^, ^8^, ^9^, ^10^ One of the most basic and limiting bottlenecks revolves around procurement and supply chain deficiencies that result in stockouts of key supplies at point of care.^11^ It is not enough to provide resources for medical supplies; these supplies have to be in the right place at the right time, consistently and reliably.^11^, ^12^ Additionally, organisation of services is often suboptimal. For any antenatal care visit, the required set of health interventions is often delivered at several physical locations and requires multiple appointments, resulting in long waiting times and several visits to the facility.^10^

**Research in context**

**Evidence before the study**

Maternal and newborn mortality and morbidity remain unacceptably high in many low-resource settings. A substantial proportion of these adverse outcomes are preventable as several interventions have been shown to be effective and supported by high-quality evidence. This issue is particularly relevant during the antenatal period, since in many low-resource settings antenatal care visits represent the only opportunity for providing pregnant women with health-care services. However, stockouts of medical supplies at the point of care often prevent implementation of these services. The distribution of supply kits targeted at women, providers, or health facilities has been suggested as a strategy to increase the adoption of evidence-based practices. However, the bulk of the evidence on the effectiveness of these kits derives from observational studies and has focused mainly on clean childbirth and reducing the incidence of infection and its complications, particularly in settings where women give birth at home. Despite the important role of antenatal care and the potential value of supply kits, to the best of our knowledge the delivery of supply kits at point of care to improve the quality of antenatal care visits has not been formally tested. The design of this trial was therefore based on the results of formative research that aimed to understand the barriers that hinder health practitioners' ability or willingness to implement evidence-based antenatal care practices and on a national needs assessment of maternal and neonatal health.

**Added value of the study**

The results of our stepped-wedge, cluster-randomised controlled trial show that the provision, at point of care, of supplies for evidence-based practices packaged in kits resulted in a vast improvement in the quality of antenatal care compared with the routine system for procurement and distribution of supplies used by the Ministry of Health of Mozambique. The effect was found to be immediate and sustained over time, with negligible heterogeneity between sites. The intervention improved care for the main drivers of maternal and infant morbidity and mortality, including hypertensive disorders of pregnancy, anaemia, and maternal infections. The combination of a robust design, large sample size, and large effect size provides a strong level of evidence to support the effectiveness of the intervention, since bias or random error are unlikely to explain these findings. This was a pragmatic trial designed from the ground up to inform policy makers. The study population included all women attending antenatal care visits and the intervention was delivered by available health-care workers under routine care conditions by use of the government antenatal care registration logbooks as a source of data.

**Implications of all the available evidence**

Policy makers should consider the use of supply kits to efficiently provide medical supplies at the right time, consistently, and reliably in settings where supply chain deficiencies and stockouts are major barriers to health care.

With a maternal mortality ratio of 489 maternal deaths per 100 000 livebirths in 2015, only 54% of births attended by a skilled birth attendant, and 51% of pregnant women receiving four or more antenatal care visits,^1^, ^13^ Mozambique needs to develop and prioritise strategies to increase contact of pregnant women with the health system and to improve the quality and integration of antenatal care services. To explore deficiencies and potential solutions for improving antenatal care in Mozambique, WHO and the Mozambican Ministry of Health launched a two-pronged study in 2010. The formative research component of the study assessed factors affecting the implementation of evidence-based antenatal care services.^8^, ^14^ The findings of this study were then used to inform the development of an intervention to improve delivery of antenatal care, to be assessed through a subsequent randomised controlled trial.^14^

The methods and results of the formative research have been published elsewhere.^8^, ^14^ This research identified deficient infrastructure and poor functioning of the supply chain system—resulting in absence or scarcity of supplies—as major barriers to the delivery of antenatal care services, with a strong recommendation to strengthen supply chain functionality through the introduction of a kit system for supplying essential antenatal care commodities to health providers.^8^

We compared a supply chain strategy based on the provision of medical supply kits with the conventional procurement and distribution procedures used by the Ministry of Health in Mozambique, with respect to the delivery of practices for the detection, treatment, and prevention of major maternal and perinatal health-related conditions.

## Methods

### Study design

We did a pragmatic, facility-based, cluster randomised controlled trial with a stepped-wedge design in which participating antenatal care clinics were randomly assigned to shift from control to the intervention on prespecified dates. After 3 months of baseline data collection in all clinics, the intervention was rolled out sequentially to a new clinic every 2 months (length of the step). The trial lasted 23 months, including the 3 months of baseline data collection. The methods of the study are described in detail in the study protocol.^14^

### Participants

Clusters were antenatal care clinics in health facilities selected purposely by the Ministry of Health according to its programmatic activities and priorities and with geographical representation of the three regions of Mozambique (north, centre, and south). Antenatal care clinics were eligible if they were not already implementing the proposed antenatal care package; they served at least 200 new pregnant women per year; they had Maternal and Child Health (MCH) nurses; and they were willing to participate. All women attending antenatal care visits at the participating clinics were included in the trial. The ten participating clinics have been described in the study protocol^14^ and are listed in the appendix. All clinics were Ministry of Health facilities and provided antenatal care free of charge under the public health system in which patients do not pay for maternal and child health care. All clinics provided the same standard of care in accordance with national Ministry of Health guidelines for antenatal care at health centre level. Two of the clinics (3-Songo and 5-Matola) received some additional resources from non-governmental partners.

### Randomisation

Clinics were assigned to one of ten start dates by the study statistician via a computer-generated list of random numbers. Concealment of the intervention start date was not possible for logistic reasons, because of the involvement of the Ministry of Health in the preparatory activities required to launch the intervention at each clinic.

### Procedures

The intervention was multifaceted with four components described in the panel, which pertain to the cluster level: antenatal care kits (boxes containing supplies necessary to carry out a specific number of antenatal care visits); a cupboard to organise and store the supplies locked in the antenatal care room; a tracking sheet to monitor the kits' stock levels; and a training session for the health-care providers at the beginning of the intervention.

Panel **Components of the intervention**

**Component 1: antenatal care kits containing the necessary medicines, laboratory supplies, materials, and equipment**

Four different kits were designed. Each health facility was provided with the antenatal care kits, which include the commodities required for first and follow-up antenatal care visits in accordance with Ministry of Health guidelines.

**Component 2: cupboard**

A cupboard for storage of the kits was provided in the room where the antenatal care visits took place, which allowed the nurses to have easy and quick access to all necessary materials during the antenatal care visit, and ensured secure storage of items.

**Component 3: tracking sheet**

A tracking sheet was introduced to monitor use of kits and stock levels to avoid stockouts.

**Component 4: training session**

At the start of the intervention in each site, a refresher course on the essential interventions for antenatal care was held with all the Maternal and Child Health (MCH) nurses in the health facilities involved in delivering antenatal care, as well as the pharmacist and laboratory technician. These nurses were also trained in how to use the contents of the kit, such as the proteinuria test and blood pressure measurement, and in procedures to ensure continuous supply of the medicines and materials needed.

The kits were designed by two experts in procurement and supply chain management on the basis of the evidence-based antenatal care interventions in the national guidelines (see the appendix for the list of interventions). Because of the importance of the first antenatal care visit, the trial differentiated between first and follow-up antenatal care visits (visits other than the first). We designed four types of kits: Kit A contained supplies necessary to carry out 100 first antenatal care visits; Kit B contained supplies necessary to carry out 200 follow-up antenatal care visits; Kit C contained urine collection containers; and Kit D contained long-lasting impregnated bednets. For this trial, Kit A and Kit B were procured in the Netherlands and imported as ready-made boxes in accordance with national regulatory importing procedures. Kit C was purchased in Mozambique, and Kit D and antiretroviral drugs were provided through the Ministry of Health. The International Centre for Reproductive Health in Mozambique, the local implementing partner, was responsible for importation, storage, and distribution of the kits. Automatic sphygmomanometers with rechargeable batteries were part of the intervention but were not packed inside the kits because they were a one-time delivery. The full list of the products contained in Kits A and B, and photos of the kits, can be found in the appendix.

Data collection started in March, 2014, in all antenatal care clinics. The first clinic entered the intervention in June, 2014, and the last in December, 2015. The dates of the launches of the intervention in each facility are shown in the appendix. Before the day of the launch, the local coordination team would be deployed to the clinic for preparation of the intervention. The kits were received, stored, and organised, the cupboard for storage was set up, and the antenatal care room or rooms were rearranged to accommodate changes in patient flow required by the introduction of the intervention. The training session was carried out with attendance of all MCH nurses involved in antenatal care services at the clinic, as well as laboratory and pharmacy technicians. The content of the training is outlined in the panel. On the day of the launch, the nurses would start by addressing the awaiting pregnant women to explain the new system of kits and their contents, the antenatal care practices they were going to receive, the importance of these practices, and the advantages expected. This process was implemented ten times—before the launch of the intervention in each facility.

An official from the Ministry of Health was actively involved in every step of the trial, including the launches and the monitoring visits. Nurses were supervised by the Ministry of Health in the context of their routine monitoring activities.

During the control period, each clinic provided antenatal care according to standard practice and functioned under the regular procurement system. The components of the antenatal care visits were the same in both groups, as per national guidelines (appendix). The kits contained, among other items, rapid tests for HIV, syphilis, haemoglobin, and proteinuria. Rapid tests for HIV and syphilis were already a routine part of antenatal care services before the intervention, whereas proteinuria and haemoglobin tests were only done at the laboratory.

### Outcomes

Ten practices for women attending the first antenatal care visit were initially targeted by the intervention and included in the protocol as primary outcome candidates.^14^ The protocol prespecified that three among those ten practices would be selected as primary outcomes, on the basis of the analysis of outcome delivery rates with data collected before the implementation of the intervention (ie, in step 1). This analysis was done during step 2 and the results are presented in the appendix. From the initial list of outcomes, iron/folic acid supplementation and tetanus toxoid administration were excluded before the analysis was done because the information required to compute these variables was not recorded in the antenatal care logbook. Screening for hypertensive disorders was split into its two elements: screening for high blood pressure and screening for proteinuria. These changes led to the final list of nine outcomes that were evaluated for the first antenatal care visits. The full list of outcomes and definitions is provided in the appendix. Among these nine practices, the three with the lowest delivery rate during step 1 were selected as primary outcomes: screening for proteinuria, screening for anaemia, and treatment of parasitic worms with mebendazole. The other six practices (screening for high blood pressure, preventive treatment for malaria, screening for HIV, treatment for HIV, screening for syphilis, and treatment for syphilis) were selected as secondary outcomes.

Secondary outcomes also included delivery of antenatal care practices in follow-up visits. These practices are described in the appendix. A composite outcome was also prespecified in the protocol.

All outcomes were measured with routine data extracted from the antenatal care logbook. Antenatal care nurses are required to register all antenatal care visits and practices delivered in each visit in standardised logbooks provided by the Ministry of Health (appendix). Although the Ministry of Health compiles data from monthly summaries generated by the nurses on the basis of the data in the logbooks, no system is in place for systematic digital storage of this information. Women do not have clinical records, and the logbook was the only source document for the trial. Data management procedures were developed and implemented in all ten participating antenatal care clinics with the purpose of transferring data in the logbook to the data management centre. Nurses were trained in how to complete the logbooks correctly by use of the coding system. Research assistants hired for the trial regularly reviewed the logbooks in each antenatal care clinic, took digital photos of each page of the logbook, and sent them to the data management centre in Maputo. We originally intended to link first and follow-up antenatal care visits for each woman by assigning a unique study participant ID at enrolment; however, implementation of such a system was challenging in practice and it is only available for a subset of the data. Data management and quality control procedures have been described in the protocol^14^ and are summarised in the appendix. These procedures included several data quality audits and monitoring activities.

### Statistical analysis

The Ministry of Health provided the number of antenatal care visits in each selected clinic for 2011, which was used to calculate the sample size, as reported in the study protocol.^14^ We assumed, conservatively, a baseline frequency of 30% for each selected health practice, and an increase to 60% with implementation of the intervention. For a 0·05 alpha level, 80% power, and an intra-cluster correlation coefficient of 0·05, six clusters were needed. To protect against pre-randomisation exclusions and dropouts, we decided to include ten clusters (antenatal care clinics). All sites completed the study and it is therefore overpowered for these initial assumptions.

Analyses were done according to the intention-to-treat principle.^15^ Because we prespecified three primary outcomes, the level of significance was adjusted for multiple comparisons and set to 0·016 rather than 0·05. For dichotomous outcomes, a multilevel logistic regression was used to estimate odds ratios [OR] and 99% CI for exposure to the intervention across datapoints during the intervention period compared with the pre-intervention period. For the composite outcome score, mean differences and 99% CI were computed by use of multilevel linear regression.^15^ The clinic was entered in these models as a random effect. Effect sizes were adjusted for time trends by including time in the model as a fixed effect.

This trial is registered with the Pan African Clinical Trial Registry, number PACTR201306000550192.

### Role of the funding source

The funders of the study had no role in study design, data collection, data analysis, data interpretation, or writing of the report. The corresponding author, APB, EB, SG, MHN, and SM had full access to all the data in the study. All authors had final responsibility for the decision to submit for publication.

## Results

**Figure 1:**
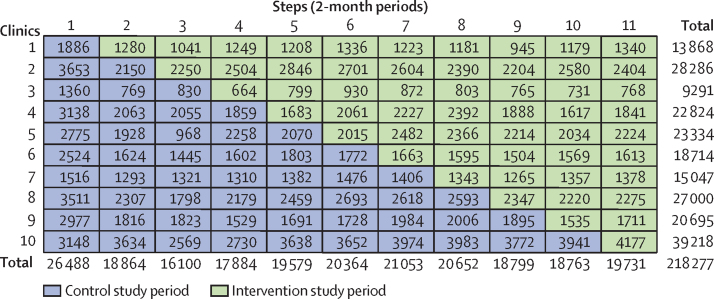
Trial diagram showing number of antenatal care visits by antenatal care clinics and steps

Between March, 2014, and January, 2016, 218 277 eligible antenatal care visits were registered in the logbooks at the ten clinics participating in this trial (68 598 first visits and 149 679 follow-up visits). No losses or exclusions of study facilities occurred. Figure 1 shows the distribution of antenatal care visits by clinic and step. The distribution stratified by the first visit and follow-up visits is shown in the appendix. The sample size was larger for step 1 because this step comprised a 3-month period (baseline data collection), compared with a 2-month period for all subsequent steps. Furthermore, clinic 10 was the largest and spent most of the trial in the control group; as a consequence, the control period has a higher number of antenatal care visits than the intervention period (figure 1; table 1).

**Table 1:** Population characteristics, by study period

		**Control period (n=122 884)**	**Intervention period (n=95 393)**
First visit		37 826 (30·8%)	30 772 (32·3%)
Fourth visit		11 319 (9·2%)	8635 (9·0%)
Other follow-up visits		73 739 (60·0%)	55 986 (58·7%)
First antenatal care visit			
	Women screened	37 826	30 772
	Age, years	24·0 (6·1)	24·1 (6·2)
	Gestational age, weeks	22·9 (6·7)	22·4 (6·7)
Follow-up antenatal care visits			
Women screened		85 058	64 621
Age, years		23·9 (6·0)	24·0 (6·0)
Gestational age, weeks		30·4 (6·5)	29·6 (6·5)

Data are n (%) or mean (SD).

Women's characteristics and antenatal care visit characteristics did not change over time. The distribution of the first and follow-up antenatal care visits, participants' ages, and gestational ages were similar between the control and intervention periods (table 1).

Deployment of the intervention was delayed by 1 month in the first antenatal care clinic, which resulted in a 3-month baseline period. There were no other delays in rollout of the intervention. All ten clinics received the intervention as described in the protocol, with only minor deviations. The tracking sheet was simplified to only monitor stock levels of the kits rather than the various products inside them. The training session at the launch of the intervention in each clinic lasted 1 day rather than 3 days as originally planned because 1 day was deemed sufficient. During the intervention period, two clinics had a single 3-day period with no kits. There were a few isolated issues with expiry of HIV and syphilis tests; these issues were short lasting and were overcome by the health facility providing routine supplies while awaiting arrival of new kits.

Table 2 shows the intervention effect sizes for each outcome. Clinically and statistically significant improvements in all three primary outcomes were observed. 5519 (14·6%) of 37 826 women were screened for anaemia (the first primary outcome) in the control period, compared with 30 057 (97·7%) of 30 772 women in the intervention period (adjusted OR 832·40; 99% CI 666·81–1039·11; p<0·0001; table 2). 3739 (9·9%) of 37 826 women were screened for proteinuria (the second primary outcome) in the control period, compared with 29 874 (97·1%) of 30 772 women in the intervention period (adjusted OR 1875·18; 99% CI 1447·56–2429·11; p<0·0001; table 2). 17 926 (51·4%) of 34 842 women received mebendazole for treatment of parasitic worms (the third primary outcome) in the control period, compared with 24 960 (88·2%) of 28 294 women in the intervention period (adjusted OR 1·88; 99% CI 1·70–2·09; p<0·0001; table 2).

**Table 2:** Effect of the intervention during first antenatal care visits for primary and secondary outcomes

	Control period*	Intervention period*	**Mixed-model adjusted odds ratio of intervention effect (99% CI)**†	**p value**	**ICC**‡
**Primary outcomes**					
Screening for anaemia	5519/37 826 (14·6%)	30 057/30 772 (97·7%)	832·40 (666·81–1039·11)	<0·0001	0·588
Screening for proteinuria	3739/37 826 (9·9%)	29 874/30 772 (97·1%)	1875·18 (1447·56–2429·11)	<0·0001	0·351
Treatment for worms (mebendazole)	17 926/34 842 (51·4%)	24 960/28 294 (88·2%)	1·88 (1·70–2·09)	<0·0001	0·291
**Secondary outcomes**					
Screening for high blood pressure	24 654/37 826 (65·2%)	30 487/30 772 (99·1%)	609·29 (466·69–795·46)	<0·0001	0·584
Preventive treatment for malaria	12 725/19 844 (64·1%)	14 373/15 350 (93·6%)	3·68 (3·17–4·28)	<0·0001	0·119
Screening for HIV	33 756/35 284 (95·7%)	27 573/28 430 (97·0%)	1·04 (0·84–1·27)	0·662	0·020
Treatment for HIV	2396/2678 (89·5%)	1622/1797 (90·3%)	1·61 (0·98–2·65)	0·013	0·088
Screening for syphilis	24 833/37 826 (65·7%)	29 385/30 772 (95·5%)	23·50 (20·56–26·86)	<0·0001	0·083
Treatment for syphilis	672/1106 (60·8%)	696/807 (86·2%)	2·49 (1·38–4·51)	0·0001	0·024

Data are n/N (%), unless otherwise indicated.

* Denominators vary according to the population eligible for each outcome as described in the appendix.

† Mixed-model odds ratios account for the clustering of patients within clinics and adjust for time trends. Under the stepped-wedge design, the adjusted odds ratios are calculated with the use of all data points in the intervention period versus the control period and therefore represent the average odds of exposure to the intervention.

‡ Intracluster correlation coefficient (ICC) during the control period.

Clinically and statistically significant improvements were also observed in four of the six secondary outcomes evaluated in the first antenatal care visits (table 2). A small improvement was observed in HIV screening and treatment but this result was not clinically significant. The overall pattern of improvement was less striking in follow-up visits, although a statistically and clinically significant increase in practice coverage was observed in four of the six secondary outcomes evaluated in follow-up visits (appendix). The intervention was also found to have a statistically significant effect on the composite outcome score (adjusted mean difference 1·72; 99% CI 1·70–1·74; p<0·0001; appendix).

**Figure 2:**
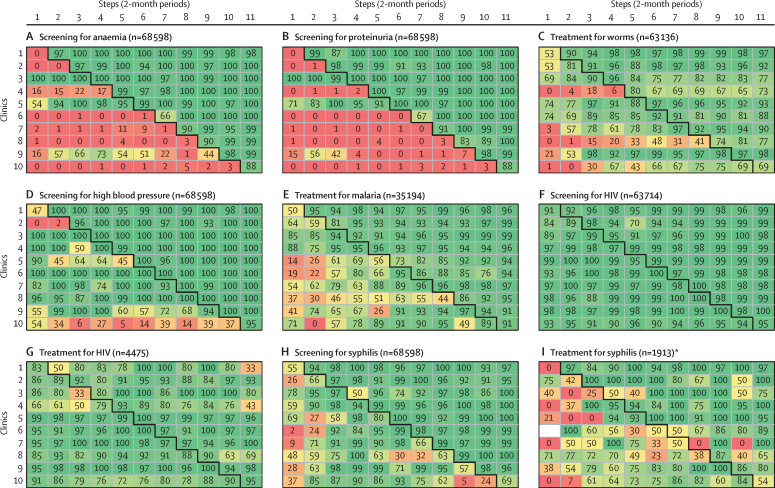
Outcome rates, by step and health facility; first antenatal care visits Each cell contains the proportion of women who received each specific antenatal care practice in the corresponding antenatal care clinics and steps. For each of the nine panels in this figure, the ten clinics are represented in the y-axis while the steps of the trial are represented in the x-axis. *When the denominator to compute the cell rate is 0, cells are coloured in white.

Practice delivery rates at the first antenatal care visits are stratified by facility and step in figure 2. These secondary analyses show that practice delivery increased immediately after the introduction of the intervention (ie, in the same step the intervention was introduced), that during the intervention period almost no heterogeneity was observed between clinics, and that once the rates increased these changes were sustained over time (figure 2). These patterns were particularly evident for screening for proteinuria and anaemia (figure 2). The same patterns were observed for follow-up visits, which are presented in the appendix.

## Discussion

The results of this stepped-wedge cluster-randomised controlled trial show that implementation of a supply chain strategy based on the provision of pre-packed supply kits at the point of care can generate major and long lasting improvements in practice delivery and thus in quality of antenatal care. Importantly, through integration of all essential antenatal care practices, the kits created the necessary conditions for a woman-centred approach, resulting in improvements in antenatal care across practices (all >80% coverage for the whole intervention period). Furthermore, when looking at the effect of the intervention in each clinic and over time, we found a uniform effect across clinics, which was immediate and sustained throughout the entire intervention period.

Our simple intervention addressed several limitations of the health system in Mozambique. Packaging of all required supplies and timely delivery of these kits at the clinics addressed weaknesses in the procurement and supply systems.^11^ With the kits, screening for proteinuria and haemoglobin switched from laboratory testing to rapid testing, which enabled nurses to screen, diagnose, and treat women in a single antenatal care session, thus optimising the scarce contact between these women and the health-care system and ensuring that these women access the full range of recommended interventions. The shortage of human resources for antenatal care results in overburdened and frustrated nurses who are unable to deliver all recommended practices.^12^, ^16^ The trial kits streamlined the antenatal care processes, achieving increased efficiency without increasing the size of the workforce.

Vertical approaches to health care, focusing on a single health condition, have been criticised because they can force busy health-care providers to deflect attention from other critical activities, which could result in deterioration of the overall quality of care.^16^, ^17^, ^18^ Baseline data from this trial are consistent with this view. Vertical, well-resourced HIV programmes are deeply established in Mozambique; we saw that screening and treatment for HIV were two practices with very high baseline coverage, at 90% or more, and therefore little room for improvement as reflected in the non-significant increase, whereas screening for high blood pressure, proteinuria, and anaemia was much less common (table 2). The public health and practical implications of this finding are important, particularly in a setting where haemorrhage and hypertensive disorders of pregnancy are leading direct causes of maternal mortality.^19^

The supply of pre-packed kits—targeted at women, health-care providers, or facilities—has been proposed as a simple, low-cost intervention with the potential to address challenges routinely encountered in low-resource settings that prevent patients from receiving appropriate care.^20^, ^21^, ^22^, ^23^ However, in maternal health, almost all evidence on supply kits derives from observational studies and has focused mainly on clean childbirth and reducing the incidence of infection and its complications, particularly in settings where women give birth at home.^20^, ^21^ To our knowledge, this is the first trial assessing supply kits targeted at health facilities as an intervention to improve coverage of routine evidence-based practices for maternal care.^24^

In this trial, the combination of a robust design, large sample size, and large effect size provides a strong level of evidence to support the effectiveness of the intervention, since bias or random error are unlikely to explain these findings. Other strengths of the study include the stepped-wedge design, which allowed delivery of the intervention in all clusters, avoiding some of the logistic and political challenges typically faced by researchers.^25^ We randomised the order in which facilities received the intervention and adjusted for potential secular trends. We also validated the source data by doing surveys with women leaving antenatal care and doing an audit. Contamination was deemed unlikely because it would imply the improbable transfer of kits between clinics, which were dispersed across the country. Additionally, the kits' stocks were monitored.

This was a pragmatic trial designed from the ground up to inform policy makers. The close partnership between the Ministry of Health and WHO, and the ownership and full engagement of the Ministry of Health, catalysed this implementation research, embedded in a real-world setting and driven by the needs and priorities of the country. Consequently, a relevant and practical intervention was designed on the basis of the formative research. The study population included all women attending antenatal care visits, with no exclusion criteria. The intervention was implemented by the available nurses under routine care conditions and regardless of any event occurring in the clinics during the trial period. We used the government antenatal care registration logbook as our source of data and thus the trial did not place additional burden on the health-care providers. To our knowledge, this is the first trial to evaluate an intervention with these characteristics.

For all parties involved in the study, scaling up and sustainability were important considerations. Subsequent to the trial, the study partners, including the Ministry of Health, have been engaged in an assessment of the cost-effectiveness and implementation options for scaling up the use of antenatal care supply kits in Mozambique. This information will inform how antenatal care supply kits could be incorporated into the supply chain in Mozambique, including whether the use of other (private) supply solutions would be feasible and cost-effective.

Our study has some limitations. The stepped-wedge design can be vulnerable to secular trends if outcomes are already improving. However, the large sample size and large number of steps in this trial allowed us to estimate and adjust for time trends.^15^ The effect might also be explained by differences in reporting during the intervention period. To avoid this problem, we isolated the data collection procedures from those relating to implementation of the interventions as much as was feasible and implemented strict data management procedures that included several data audits.

Although rapid tests for HIV and syphilis were being used in antenatal care before the intervention, the kits introduced two additional rapid tests for proteinuria and anaemia—the conditions for which the most substantial increase in screening was observed. The availability of rapid tests for these conditions is likely to have an effect independently of the provision of kits.^26^ Unfortunately, however, in evaluations of complex interventions delivered as a package, disentangling the individual effect of each component is not possible.

We could not report on all the initially prespecified secondary outcomes because of limitations in the data collection instrument from the Ministry of Health. Iron and folic acid supplementation and tetanus toxoid administration had to be excluded because the information to compute these outcomes was not recorded in the logbook. Lastly, we did not collect data on final maternal and newborn outcomes, so the overall effect of the antenatal care practices on mortality or morbidity cannot be assessed. However, we only included evidence-based practices for which the benefits have already been clearly established.

The health-system and resource challenges underpinning the design of our intervention are undoubtedly not unique to the facilities participating in this trial or to Mozambique.^11^, ^12^ This study provides crucial information to policy makers in countries with similar supply chain deficiencies but its relevance goes well beyond this obvious target. We believe that this simple intervention could be easily adapted beyond low-resource settings and beyond the area of maternal health. The simplicity and the nature of the intervention make it likely to be acceptable and adaptable across many countries and settings. However, context-specific kits need to be designed. What to include, where to produce and store the kits, and how to distribute these kits to facilities will vary across countries and settings and requires careful analysis to avoid the very same challenges that these kits aim to address.

In conclusion, although the evidence-based practices for improving maternal and newborn health are well known, approaches to effectively and sustainably scale up these interventions are urgently needed, particularly in low-resource settings. The results of this study show that a simple intervention focused on the provision, at point of care, of supplies for evidence-based practices packaged in kits resulted in a vast improvement in quality of antenatal care when compared with the routine national system for procurement and distribution of supplies. The pragmatic nature of this trial, embedded in routine care conditions, makes these findings particularly valuable for policy makers in low-resource settings.
