# Supplementary material for: Provision of medical supply kits to improve quality of antenatal care in Mozambique: a stepped-wedge cluster randomised trial
Source: Lancet Glob Health. 2017 Dec 12;6(1):e57–65. doi: 10.1016/S2214-109X(17)30421-7 (PMC5732314; doi:10.1016/S2214-109X(17)30421-7)
Supplement: Supplementary appendix [file mmc1.pdf]

THE LANCET
Global Health

Supplementary appendix

This appendix formed part of the original submission and has been peer reviewed. We post it as supplied by the authors.

Supplement to: Betrán AP, Bergel E, Griffin Se et al, for the WHO Antenatal Care Trial in Mozambique Research Group. Provision of medical supply kits to improve quality of antenatal care in Mozambique: a stepped-wedge cluster randomised trial. *Lancet Glob Health* 2018; **6:** e57–65.

Supplementary Appendix

Provision of medical supply kits to improve quality of antenatal care in Mozambique: a stepped wedge cluster randomized trial

Table of Contents

1. Health facilities included in this trial, location and date of launch of the intervention 2
2. Components of the ANC visits as per national guidelines 3
3. Components of Kit A and Kit B 4
4. Photos of the kits 5
5. Detailed list of outcomes and definitions 6
6. Practice delivery rates at baseline (Step 1) 8
7. Data management and data quality assurance 9
8. Diagram of first and follow-up ANC visits independently 9
9. Effect of the intervention in follow-up antenatal care visits 10
10. Effect of the intervention in composite outcome 13
11. Ethical aspects of the trial 13
12. Checklist of items to be included when reporting a cluster randomized controlled trial 14
13. Data collection instrument: ANC logbook 16

1. Health facilities included in this trial, location and date of launch of the intervention

Clusters were antenatal care (ANC) clinics in health facilities. The 10 participating ANC clinics were selected purposely by the Ministry of Health (MOH) according to its programmatic activities and priorities and with geographical representation of the three regions of Mozambique (North, Center and South). Table S1 shows the health facilities listed chronologically according to the start of the intervention. It also shows the date of the launch of the intervention in each facility as well as its location.

Table S1: Health facilities listed in chronological order according to the start of the intervention. The date of the launch of the intervention in each facility is also shown as well as well as its location. In addition, the table shows the approximate number of nurses providing ANC care in each facility and the number of first ANC visits attended in 2011.

Order entered intervention	Health Center (HC)	Region and location	Setting	Number of first ANC visits attended in 2011*	Number of nurses proving ANC (approx.)	Date launch intervention
1	**HC of Anchilo**	North: Nampula Province	Rural	1894	2	June 2 2014
2	**HC N° 2**	Central: Tete City	Urban	2778	4	August 1 2014
3	**HC annexed to the rural hospital of Songo**	Central: Tete Province	Rural	1281	2	October 3 2014
4	**HC annexed to the district hospital of Dondo**	Central: Sofala Province	Rural	2728	4	November 28 2014
5	**HC da Matola II**	South: Maputo Province	Urban	3276	6	January 30 2015
6	**HC 1ro de Maio**	South: Maputo City	Urban	3165	2	April 8 2015
7	**HC annexed to the rural hospital of Chowke**	South: Gaza Province	Rural	3480	6	June 4 2015
8	**HC annexed to the rural hospital of Montepuez**	North: Cabo Delgado Province	Rural	4182	4	August 5 2015
9	**HC of Chibuto**	South: Gaza Province	Rural	9756	6	October 1 2015
10	**HC 25 de Setembro**	North: Nampula city	Urban	5960	8	December 4 2015

* Data provided by the Ministry of Health.

2. Components of the ANC visits as per national guidelines

National guidelines from the Ministry in Health of Mozambique promote an ANC model including a series of evidence-based interventions for each ANC visit. Table S2 provides the interventions to be delivered to the women by ANC visit.

Table S2: Elements/interventions in the ANC package of the Ministry of Health by ANC visit.

No.	Elements of ANC	First ANC visit	Follow-up ANC visit*
**1**	Clinical physical examination	X	X
**2**	Obstetric examination	X	X
**3**	Measurement of haemoglobin level	X	From 32 weeks
**4**	Assessing proteinuria	X	X
**5**	Measurement of blood pressure	X	X
**6**	Performance of syphilis test and treatment	X	
**7**	Prevention of anaemia		
	7.1 Deworming (mebendazole)	X	
	7.2 Ferrous sulfate + folic acid	X	X
**8**	Malaria prevention		
	8.1 ITP (Sufadoxine-pyrimethamine)	X	X
	8.2 Mosquito net	X	
**9**	HIV testing & counselling	X	3 months after first test
**10**	Vaccination	X	X
**11**	Complementary intervention: Provision of ARV treatment	X	X

* Follow-up ANC visits are visits other than the first.

3. Components of Kit A and Kit B

Table S3: Composition of Kit A

	Product	Pack size	Quantity of packs
**1**	Protein urine test strips	100	1
**2**	HIV Rapid Diagnostic Test - Determine	100	1
**3**	HIV Rapid Diagnostic Test – Unigold	20	1
**4**	Syphilis Rapid Diagnostic Test	100	1
**5**	Haemoglobin test strips	200	1
**6**	Ampoule of penicillin benzathine 2,4 MIU	50	1
**7**	Water for injection, 5ml ampoule	50	1
**8**	Syringe 5 ml with 21G needle	100	1
**9**	Mebendazole 500mg tablets	100	1
**10**	Sufadoxine-pyrimethamanine 500-25mg	150	2
**11**	Ferrous Sulphate/Folic Acid tablets (90/1)	1000	3
**12**	Small tablet bags	100	1
**13**	Latex examination gloves	100	2
**14**	Disinfectant	0.5 litre	1
**15**	Cotton wool	1 roll	1
**16**	Lancet	200	1
**17**	Chase buffers	1	2
**18**	Capillary EDTA tubes	1	3

Table S4: Composition of Kit B.

	Product	Pack size	Quantity of packs
**1**	Protein urine test strips	100	2
**2**	HIV Rapid Diagnostic Test - Determine	100	1
**3**	HIV Rapid Diagnostic Test – Unigold	20	1
**4**	Sufadoxine-pyrimethamanine 500-25mg tablets	150	4
**5**	Ferrous Sulphate/Folic Acid tablets (90/1)	1000	6
**6**	Small tablet bags	500	1
**7**	Haemoglobin test strips	200	1
**8**	Latex examination gloves	100	4
**9**	Disinfectant	0,5 litre	1
**10**	Cotton wool	1 roll	1
**11**	Lancet	200	1
**12**	Chase buffers	1	1
**13**	Capillary EDTA tubes	1	2

4. Photos of the kits

Fig S1 and S2 present photos of Kit A; a ready-made box that is easily carried.

Fig S1: Photo of an opened Kit A.

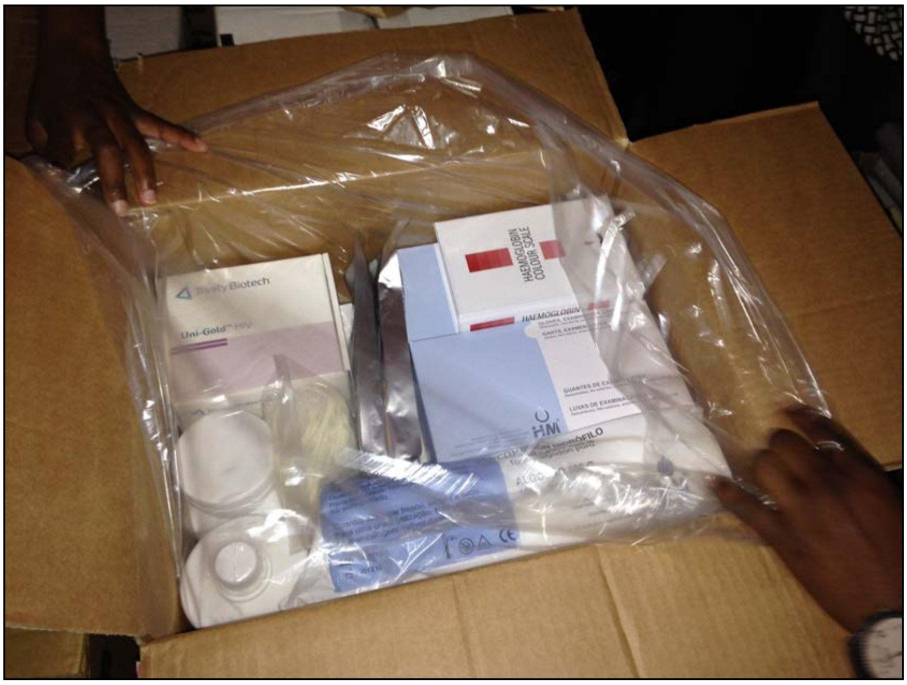

Fig S2: Photo of the kits stacked.

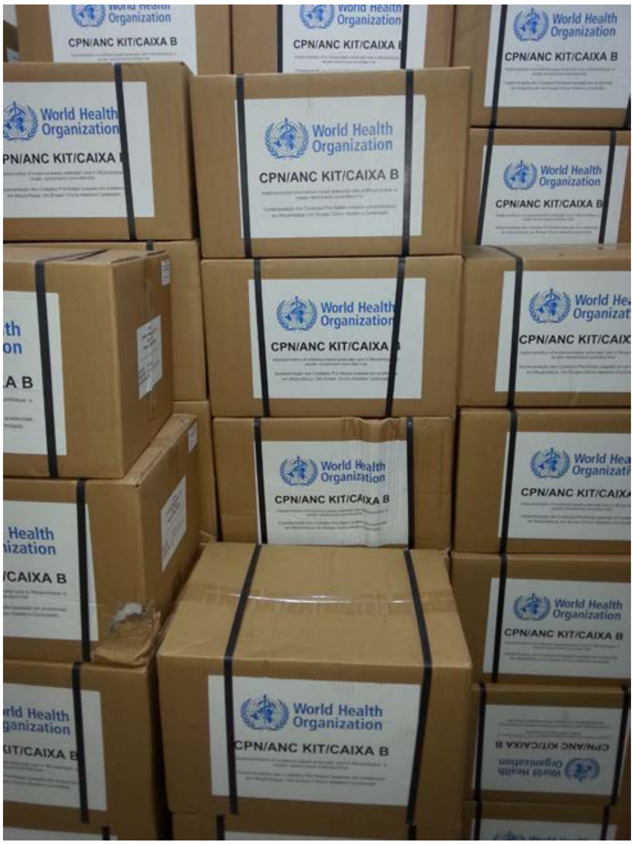

5. Detailed list of outcomes and definitions

For the purpose of the data analysis, the definitions of the outcomes had to be operationalized to be able to compute them from the data collected in the ANC logbook. For example, according to national guidelines for ANC, mebendazole should be given to women in the second or third trimester of pregnancy only. Under these guidelines, women were eligible to receive mebendazole if they had more than or equal to 12 weeks gestation and thus those at less than 12 weeks gestation were excluded from the denominator. Tables S5 and S6 list the outcomes and how they were defined and any other considerations for first and follow-up ANC visits, respectively. Section 12 of this Supplementary Appendix presents the logbook from where the data was extracted.

Table S5: Primary and secondary outcomes in first ANC visits, definitions, considerations and variables used for their operationalization and calculation within the context of the ANC logbook in Section 12 of this Supplementary Appendix.

Outcome	Definition/considerations	Eligible women (denominator)
**Screening practices**		
Blood pressure measured in 1st visit	• Proportion of eligible women who had blood pressure measured<br>• Sphygmomanometer provided as part of the intervention	All women attending the 1st ANC visit
Proteinuria assessed in 1st visit	• Proportion of eligible women who had proteinuria assessed<br>• Dipsticks provided in the ANC kits	All women attending the 1st ANC visit
Syphilis rapid test performed in 1st visit	• Proportion of eligible women who had a syphilis test performed<br>• Syphilis rapid test provided in the ANC kits	All women attending the 1st ANC visit
HIV test performed in 1st visit	• Proportion of eligible women who had the HIV test performed<br>• HIV rapid tests provided in the ANC kits (Determine® and Unigold®)	All women whose HIV status in the 1st ANC visit was negative or unknown, and were not under treatment for HIV
Haemoglobin measured in 1st visit	• Proportion of eligible women who had haemoglobin levels measured<br>• Haemoglobin colour scale tests provided in the ANC kits	All women attending the 1st ANC visit
**Treatment/prevention practices**		
Penicillin received in 1st visit (if syphilis positive)	• Proportion of eligible women who were given the 1st dose of penicillin<br>• Penicillin provided in the ANC kits	All women who had a positive syphilis test in the 1st ANC visit
Antiretrovirals received in 1st visit (if HIV positive)	• Proportion of eligible who were given antiretrovirals<br>• Antiretrovirals were not provided in the ANC kits but were part of the intervention	All women who had a positive HIV test in the 1st ANC visit
Folic acid and ferrous sulfate received in 1st visit	• All pregnant women should receive 1 iron pill per day. In practice they are given 30 pills at each appointment and are told to come back after 30 days. The fact that they received the pills is recorded in their hand-held record (caderneta); only when they receive the 3rd dose of 30 pills, it is registered in the ANC book. Thus, the logbook does not provide the information required for the analysis since it registers only the women that have received the 3rd dose (which is never in the 1st ANC visit)<br>• Folic acid/ferrous sulphate provided in ANC kit	No computed
Mebendazole received in 1st visit	• Proportion of eligible women who were given mebendazole<br>• According to the protocol of the MoH, mebendazole should only be given to women in the 2nd and 3rd trimester of pregnancy<br>• Mebendazole provided in ANC kit	All women at 13 weeks' or more gestation age, attending the 1st ANC visit

Sulphadoxine- pyrimethamine received in 1st visit	• Proportion of eligible women who were given *fansidar*<br>• According to the protocol of the MoH, *fansidar* should not be given early in pregnancy. At the beginning of the trial the norm was to start at 20 weeks. At some point during the trial this changed to 12 weeks. Conservatively, we used the 20 weeks as the cutoff throughout.<br>• *Fansidar* should not be given to women who are HIV+ (if they are taking Cotrimoxazole and/or ART)	All women at 20 weeks' or more gestation age, who are not taking cotrimoxazol and/or ART, attending the 1st ANC visit
Bed nets received in 1st visit	• The bed nets were ANC Kit D so were part of the intervention but their supply was secured through other partners, and distribution by MoH<br>• The trial only strengthened this intervention by reminding nurses in the refresher training<br>• Major stock outs are rare but there were often short delays in distribution to health facilities. In these cases, nurses would give the woman her net on her follow-up visit.	Not computed
Tetanus toxoid received in 1st visit	• The vaccine was not provided in the ANC kits<br>• Stock outs are very rare in this item<br>• What the trial did was to remind nurses in refresher training that they should vaccinate women; and in one center a cool box was provided so that they could vaccinate in all ANC rooms without having to refer the woman to another room.<br>• Women that show they have been vaccinated previously (ie. By showing vaccination card or patient hand-held record (caderneta) should not be re-vaccinated. We would not expect 100% of women to receive the vaccine.<br>• Thus, the readings from the logbook are not adequate for the analysis of this outcome	Not computed

Table S6: Secondary outcomes in follow-up ANC visits, definitions and consideration for operationalization within the context of the ANC logbook shown in Section 12 of this Supplementary Appendix.

Outcome	Definition/considerations	Eligible women (denominator)
**Screening practices**		
Blood pressure measured in follow-up visits	• Proportion of eligible women who had blood pressure measured<br>• Sphygmomanometer provided as part of the intervention	All women attending follow-up visits
Proteinuria assessed in follow-up visits	• Proportion of eligible women who had proteinuria assessed<br>• Dipsticks provided in the ANC kits	All women attending follow-up visits
Syphilis rapid test performed in follow-up visits	• Not in follow-up visits according to national guidelines	Not computed
HIV test performed in follow-up visits	• Proportion of eligible women who had the HIV test performed<br>• HIV rapid tests provided in the ANC kits (Determine® and Unigold®)	All women whose HIV status in follow-up visits was negative or unknown, and were not under treatment for HIV
Haemoglobin measured in follow-up visits	• Proportion of eligible women who had haemoglobin levels measured<br>• Haemoglobin colour scale tests provided in the ANC kits	All women attending follow-up visits
**Treatment/prevention practices**		
Penicillin received in follow-up visits (if syphilis positive)	• Not in follow-up visits according to national guidelines	Not computed
Antiretrovirals received in follow-up visits (if HIV positive)	• Proportion of eligible who were given antiretrovirals<br>• Antiretrovirals were not provided in the ANC kits but were part of the intervention	All women who had a positive HIV test in follow-up visits

Folic acid and ferrous sulfate received in follow-up visits	• All pregnant women should receive 1 iron pill per day. In practice they are given 30 pills at each appointment and are told to come back after 30 days. The fact that they received the pills is recorded in their hand-held record (caderneta); only when they receive the 3rd dose of 30 pills, it is registered in the ANC book. Thus, the logbook does not provide the information required for the analysis since it registers only the women that have received the 3rd dose (which is never in the 1st ANC visit)<br>• Folic acid/ferrous sulphate provided in ANC kit	Not computed
Mebendazole received in follow-up visits	• According to national guidelines, mebendazole is only given in the first ANC visit	Not computed
Sulphadoxine- pyrimethamine received in follow-up visits	• Proportion of eligible women who were given *fansidar*<br>• According to the protocol of the MoH, *fansidar* should not be given early in pregnancy. At the beginning of the trial the norm was to start at 20 weeks. At some point during the trial this changed to 12 weeks. Conservatively, we used the 20 weeks as the cutoff throughout.<br>• *Fansidar* should not be given to women who are HIV+ (if they are taking Cotrimoxazole and/or ART)	All women at 20 weeks' or more gestation age, who are not taking cotrimoxazol and/or ART, attending follow-up visits
Bed nets received in follow-up visits	• The bed nets were ANC Kit D so were part of the intervention but their supply was secured through other partners, and distribution by MoH<br>• The trial only strengthened this intervention by reminding nurses in the refresher training<br>• Major stock outs are rare but there were often short delays in distribution to health facilities. In these cases, nurses would give the woman her net on her follow-up visit.	Not computed
Tetanus toxoid received in follow-up visits	• The vaccine was not provided in the ANC kits<br>• Stock outs are very rare in this item<br>• What the trial did was to remind nurses in refresher training that they should vaccinate women; and in one center a cool box was provided so that they could vaccinate in all ANC rooms without having to refer the woman to another room.<br>• Women that show they have been vaccinated previously (ie. By showing vaccination card or patient hand-held record (caderneta) should not be re-vaccinated. We would not expect 100% of women to receive the vaccine.<br>• Thus, the readings from the logbook are not adequate for the analysis of this outcome	Not computed

6. Practice delivery rates at baseline (Step 1)

Table S7. Practice delivery rates among first visits in step one, which was used to define the primary outcomes of the trial.

	**n/N**	**Rate**
Screening for anaemia	1007/7792	12·9
Screening for proteinuria	951/7792	12·2
Treatment for worms (mebendazole)	2137/7225	30·1
Screening for high blood pressure	5457/7792	70·0
Preventive treatment for malaria	2089/3935	53·1
Screening for HIV	6836/7274	94·0
Treatment for HIV	562/638	88·1
Screening for syphilis	3260/7792	41·8
Treatment for syphilis	56/145	38·6

7. Data management and data quality assurance

This was a pragmatic trial which used routine data as its primary data source. ANC nurses in Mozambique are required to register all antenatal visits in standardized logbooks which are designed and provided by the MOH. While the MOH compiles data from monthly summaries generated by the nurses on the basis of the data in the logbooks, there is no system in place to systematically digitalize this information. Women have a hand-held record (caderneta) where the healthcare provider writes the information related to pregnancy. Women do not have clinical records, and the logbook was the only source document for the trial. Data management procedures were developed and implemented in all 10 participating ANC clinics with the purpose of transferring the data in the logbook to the data management center. A simple coding system was introduced for the logbooks in order to avoid the ambiguities present in the standard system for completing the logbooks, and nurses were trained in how to complete the logbooks correctly using the coding system. Research assistants hired for the trial regularly reviewed the logbooks in each ANC clinic, took digital photos of each page of the logbook and sent them to the data management center in Maputo. We originally intended to link first and follow-up ANC visits for each woman by assigning a unique study subject ID at enrolment; however, implementation of such a system was in practice challenging and it is only available for a subset of the data.

Several data quality audit and monitoring activities were implemented. Special consideration was taken to avoid bias due to changes in data quality associated with the implementation of the intervention. With this aim and to the extent possible, procedures and monitoring for data collection were deployed independently from the deployment and monitoring of the intervention.

In order to minimize potential issues with quality of the routine data collected from the logbooks, three independent data monitoring procedures were implemented. First, we conducted routine monitoring visits to verify that the trial data collection instrument –the registration logbook– was being completed appropriately and that the data management processes were being done according to standardized procedures. These visits were conducted every two to three months in both control and intervention sites. Second, once in each ANC clinic, data monitors directly observed 100 consecutive first ANC visits per facility, and registered the results of tests and procedures in a monitoring log. The data in this monitoring log was then compared to the same records in the logbooks completed by the nurses. Third, a set of surveys was conducted by interviewing a sample of 100 consecutive women at each ANC clinic as they were leaving their first ANC visit. To compare data quality between the intervention and control periods, these data surveys were conducted at three different times – at the start, middle and end of the data collection period.

Data management followed Good Clinical Practice (GCP) guidelines, and was implemented according to pre-defined HRP/WHO Standardized Operating Procedures (SOPs) for managing clinical trials. A more detailed description of these procedures can be found in the study protocol [11].

8. Diagram of first and follow-up ANC visits independently

Table S8: Trial diagram for first ANC visits. Each step equals 2 months except for step 1 which was 3 months. The ANC clinics are listed in Table S1 of this Supplementary Appendix.

	Steps											
**Clinics**	1	2	3	4	5	6	7	8	9	10	11	**Total**
1	801	615	490	617	624	719	562	593	447	515	733	6716
2	998	613	703	836	879	769	728	644	717	934	906	8727
3	275	184	164	195	210	208	183	168	162	193	185	2127
4	1071	618	708	834	486	633	631	686	529	451	546	7193
5	793	441	283	603	566	182	698	678	755	831	761	6591
6	615	333	330	448	391	358	357	324	362	394	418	4330
7	412	305	370	392	377	408	470	414	349	392	389	4278
8	1285	979	962	905	950	1107	979	938	759	927	1055	10846
9	710	501	340	458	475	423	513	425	415	449	432	5141
10	832	946	911	1008	1381	1448	1124	1149	1209	1292	1349	12649
**Total**	7792	5535	5261	6296	6339	6255	6245	6019	5704	6378	6774	68598

Control Study Period   Intervention Study Period

Table S9: Trial diagram for follow-up visits. Each step equals 2 months except for step 1 which was 3 months. The ANC clinics are listed in Table S1 of this Supplementary Appendix.

	Steps											
**Clinics**	1	2	3	4	5	6	7	8	9	10	11	**Total**
1	1085	665	551	632	584	617	661	588	498	664	607	7152
2	2655	1537	1547	1668	1967	1932	1876	1746	1487	1646	1498	19559
3	1085	585	666	469	589	722	689	635	603	538	583	7164
4	2067	1445	1347	1025	1197	1428	1596	1706	1359	1166	1295	15631
5	1982	1487	685	1655	1504	1833	1784	1688	1459	1203	1463	16743
6	1909	1291	1115	1154	1412	1414	1306	1271	1142	1175	1195	14384
7	1104	988	951	918	1005	1068	936	929	916	965	989	10769
8	2226	1328	836	1274	1509	1586	1639	1655	1588	1293	1220	16154
9	2267	1315	1483	1071	1216	1305	1471	1581	1480	1086	1279	15554
10	2316	2688	1658	1722	2257	2204	2850	2834	2563	2649	2828	26569
**Total**	18696	13329	10839	11588	13240	14109	14808	14633	13095	12385	12957	149679

Control Study Period   Intervention Study Period

9. Effect of the intervention in follow-up antenatal care visits

We originally intended to link first and follow-up ANC visits for each woman by assigning a unique study subject ID at enrolment; however, implementation of such a system was in practice challenging and it is only available for a subset of the data. In the analysis of follow-up ANC visits, women may contribute with more than one visit (i.e. repeated visits), and as such these observations are not independent. Since we were only able to identify repeated visits in a subset of women by using the unique subject ID, the main analysis was conducted without adjusting for repeating visits. However, a sensitivity analysis was conducted in this subset of women, including subject ID in the regression models as a random variable to assess the impact of the lack of independence of multiple observation on inference estimates. The impact of these adjustments on the precision of the estimates was very small. These analyses were computed by fitting generalized linear mixed models with the lme4 statistical package in R (version 3.1).

Table S11 shows the effect of the intervention in secondary outcomes in follow-up visits. A statistically and clinically significant increase in practice coverage was observed in four of the six secondary outcomes evaluated in follow-up visits. Table S11 shows that 4015 of 85058 women (4·7%) were screened for anemia in the control period, as compared to 48319 of 64621 women (74·8%) in the intervention period (adjusted odds ratio, 140·97; 99% CI, 123·60 to 160·79; P<0·001). For proteinuria, 2804 of 85058 women (3·3%) were screened for proteinuria in the control period, as compared to 59517 of 64621 women (92·1%) in the intervention period (adjusted odds ratio, 160·20; 99% CI, 136·37 to 188·19; P<0·001). For blood pressure, 57697 of 85058 women (67·8%) had their blood pressure measured in the control period, as compared to 64001 of 64621 women (99·0%) in the intervention period (adjusted odds ratio, 475·61; 99% CI, 398·70 to 567·35; P<0·001). Preventive treatment for malaria (IPT) was provided to 35254 of 68045 women (51·8%) in the control period, as compared with 33610 of 52141 women (64·5%) in the intervention period (adjusted odds ratio, 1·37; 99% CI, 1·30 to 1·45; P<0·001).

For HIV practices in follow-up visits, the intervention did not have an effect. Table S11 shows that 522 of 75017 women (0·7%) was screened for HIV in the control period, as compared to 349 of 58009 women (0·6%) in the intervention period (adjusted odds ratio, 1·24; 99% CI, 0·90 to 1·70; P=0·005). Lastly, 30 of 54 HIV-positive women (55·6%) was treated with ARV in the control period, as compared to 15 of 25 women (60%) in the intervention period (adjusted odds ratio, 1·36; 99% CI, 0·25 to 7·34; P=0·068).

The overall pattern of improvement is less dramatic in follow-up visit than in first visits. This is thought to be due to less clear national guidelines and protocols referring to follow-up visits. HIV repeat screening is only tested in some cases and HIV treatment continues at each visit.

Table S10. Effect of the intervention during follow-up antenatal care visits.

	Control period		Intervention period		Mixed Model Adjusted Odds Ratio of Intervention Effect (99% CI) ‡			P Value	ICC §
	n/N	Rate	n/N	Rate					
**Screening for anaemia**	4015/85058	4·7	48319/64621	74·8	140·97	123·60	160·79	0·0000	0·316
**Screening for proteinuria**	2804/85058	3·3	59517/64621	92·1	160·20	136·37	188·19	0·0000	0·210
**Screening for high blood pressure**	57697/85058	67·8	64001/64621	99·0	475·61	398·70	567·35	0·0000	0·540
**Preventive treatment for malaria**	35254/68045	51·8	33610/52141	64·5	1·37	1·30	1·45	0·0000	0·059
**Screening for HIV**	522/75017	0·7	349/58009	0·6	1·24	0·90	1·70	0·0851	0·005
**Treatment for HIV**	30/54	55·6	15/25	60·0	1·36	0·25	7·34	0·6412	0·068

‡ Mixed model odds ratios account for the clustering of patients within clinics and adjust for time trends. Under the stepped-wedge design, the adjusted odds ratios are calculated with the use of all data points in the intervention period versus the control period and so represent the average odds of exposure to the intervention.

§ Intracluster correlation coefficient during the control period

Figure S3. Outcome rates, by step and ANC clinic; follow-up ANC visits. Each cell contains the proportion of women who received each specific practice in the corresponding ANC clinic and step.

Steps (2 month periods)

A Screening for Anaemia (N=148 679)

Clinics	1	2	3	4	5	6	7	8	9	10	11
1	0	98	98	100	95	100	100	100	99	99	99
2	0	0	98	99	100	100	100	100	98	100	100
3	100	100	100	100	100	100	100	100	100	100	100
4	20	6	25	27	64	63	75	95	99	84	77
5	0	0	0	0	3	29	38	38	27	38	28
6	0	0	1	0	0	0	65	100	100	99	100
7	0	1	2	5	1	2	1	28	2	8	8
8	0	1	0	0	3	0	0	2	94	41	66
9	0	0	0	0	0	0	0	1	1	41	5
10	0	0	0	1	0	2	2	3	2	1	70

B Screening for proteinuria (N=149 679)

Clinics	1	2	3	4	5	6	7	8	9	10	11
1	0	97	89	99	95	100	100	100	99	96	100
2	0	1	97	99	100	100	98	99	100	97	100
3	100	100	100	100	100	100	100	100	100	100	100
4	0	0	0	3	71	88	87	98	99	98	96
5	0	0	0	0	4	80	92	88	97	98	97
6	0	0	0	0	0	0	64	100	99	100	100
7	0	0	0	0	0	1	0	42	95	100	92
8	0	0	0	0	2	0	0	2	92	77	96
9	0	0	0	0	0	0	0	1	1	64	80
10	0	0	0	1	0	1	2	1	2	1	72

C Screening for high blood pressure (N= 149 679)

Clinics	1	2	3	4	5	6	7	8	9	10	11
1	50	99	96	100	94	100	100	100	100	99	100
2	1	2	97	100	100	100	100	100	100	99	100
3	100	100	100	100	100	100	100	100	100	100	100
4	100	99	65	100	95	98	97	100	100	99	98
5	100	100	100	97	28	100	97	100	100	100	100
6	100	98	99	100	100	100	100	100	100	100	100
7	83	100	100	71	100	94	96	100	100	100	100
8	69	90	94	99	100	100	99	100	100	98	100
9	87	58	99	72	52	62	99	99	99	100	99
10	60	33	4	26	3	8	30	16	42	41	95

D Treatment for malaria (120 186)

Clinics	1	2	3	4	5	6	7	8	9	10	11
1	36	85	83	87	79	80	88	90	88	88	84
2	53	61	75	77	66	60	64	55	52	59	63
3	38	48	42	58	54	41	47	44	38	68	54
4	52	49	57	47	55	51	48	42	40	48	44
5	6	19	61	70	42	80	83	86	75	67	73
6	18	20	52	67	42	39	52	49	56	49	34
7	54	65	50	54	56	41	64	72	86	67	83
8	69	67	79	87	81	73	78	77	78	79	67
9	28	47	56	54	31	63	56	53	40	52	53
10	44	0	40	70	68	73	63	63	37	70	71

E Screening for HIV (N=133 026)

Clinics	1	2	3	4	5	6	7	8	9	10	11
1	4	3	1	2	1	1	1	0	0	0	0
2	2	3	1	2	2	0	3	0	1	0	0
3	0	1	0	0	2	1	0	0	1	0	0
4	0	0	0	0	0	1	0	0	1	1	1
5	0	0	0	0	2	0	0	0	0	0	0
6	0	1	0	1	0	0	0	0	0	0	0
7	0	0	0	1	0	1	0	1	1	0	0
8	1	0	0	0	0	0	0	0	0	0	1
9	0	0	0	0	0	0	0	0	0	0	0
10	3	1	1	2	2	1	2	1	0	1	1

F Treatment for HIV (N=79)*

Clinics	1	2	3	4	5	6	7	8	9	10	11
1				50		0	0		100		
2	67		100	100	100	0	67				
3		100			100						
4	67					0		100	50	100	
5					100						
6	100			100			100				
7				100				0			50
8	100	0						0			
9										100	
10	50	0	50	25	50	0	57	50	100		100

*When the denominator to compute the cell rate is 0, cells are coloured in white.

10. Effect of the intervention in composite outcome

We computed this score for first ANC visits and included practices targeting all or most women as opposed to practices that are intended for a small subgroup such as treatment for syphilis or HIV. To compute this score, one point was added for each intended screening or treatment intervention delivered during the ANC visits. The practices included in the score were screening for high blood pressure, proteinuria, anaemia, syphilis and HIV, and treatment for parasitic worms. These add up to a maximum score value of six points.

The intervention was also found to have a statistically significant effect on the composite outcome score (adjusted mean difference 1·72; 99% CI 1·70–1·74; p<0·0001).

Figure S4. Composite outcome score, by step and ANC clinic; first ANC visits.

	Steps										
**Clinics**	**1**	**2**	**3**	**4**	**5**	**6**	**7**	**8**	**9**	**10**	**11**
**1**	2.4	5.7	5.7	5.9	5.8	5.9	5.9	5.9	5.9	5.8	5.9
**2**	1.6	2.3	5.7	5.7	5.4	5.6	5.7	5.8	5.6	5.7	5.7
**3**	5.2	5.6	5.7	5.2	5.5	5.3	5.4	5.6	5.6	5.5	5.6
**4**	2.7	3.0	2.8	3.1	5.6	5.4	5.4	5.5	5.5	5.5	5.6
**5**	4.4	4.1	5.1	5.3	4.7	5.8	5.7	5.6	5.6	5.6	5.7
**6**	2.4	2.7	3.5	3.4	3.6	3.7	4.9	5.5	5.6	5.5	5.6
**7**	1.8	3.1	3.5	3.1	3.5	3.7	3.3	5.5	5.7	5.6	5.6
**8**	2.4	2.5	2.6	3.2	3.0	2.7	2.6	3.0	5.3	5.6	5.7
**9**	2.2	4.1	4.8	4.6	4.0	3.6	3.7	3.5	3.8	5.7	5.7
**10**	1.8	2.1	2.1	2.7	2.3	2.6	2.7	2.4	2.1	2.2	4.9

11. Ethical aspects of the trial

This protocol has been approved by the *Comité Nacional de Bioéticapara a Saúde* of the Ministry of Health in Mozambique. In addition, it has been approved by the Research Project Review Panel of the UNDP/UNFPA/UNICEF/WHO/World Bank Special Programme of Research, Development and Research Training in Human Reproduction at the Department of Reproductive Health and Research of WHO, and the WHO Research Ethics Review Committee, Geneva, Switzerland.

A waiver of consent forms for participating women was obtained since this study is considered of minimal risk (risks of daily life, and includes the risks associated with routine physical examinations and review of medical records) and it is developed in the context of regular prenatal care and using as research records those used in health care process (no data collection tools have been developed to gather data regarding women´s health for the study). Recommendations provided for The Ottawa Statement on the Ethical Design and Conduct of Cluster Randomized Trials were taken into account in the design of this trial.

12. Checklist of items to be included when reporting a cluster randomized controlled trial

Section/Topic	Item No	Standard Checklist item	Extension for cluster designs	Page No.
**Title and abstract**				
	1a	Identification as a randomised trial in the title	Identification as a cluster randomised trial in the title	1
	1b	Structured summary of trial design, methods, results, and conclusions (for specific guidance see CONSORT for abstracts)		5
**Introduction**				
**Background and objectives**	2a	Scientific background and explanation of rationale	Rationale for using a cluster design	8-9
	2b	Specific objectives or hypotheses	Whether objectives pertain to the cluster level, the individual participant level or both	8-9
**Methods**				
**Trial design**	3a	Description of trial design (such as parallel, factorial) including allocation ratio	Definition of cluster and description of how the design features apply to the clusters	8-9
	3b	Important changes to methods after trial commencement (such as eligibility criteria), with reasons		-
**Participants**	4a	Eligibility criteria for participants	Eligibility criteria for clusters	8-9
	4b	Settings and locations where the data were collected		8-9<br>Suppl. Appendix Section 1
**Interventions**	5	The interventions for each group with sufficient details to allow replication, including how and when they were actually administered	Whether interventions pertain to the cluster level, the individual participant level or both	9
**Outcomes**	6a	Completely defined pre-specified primary and secondary outcome measures, including how and when they were assessed	Whether outcome measures pertain to the cluster level, the individual participant level or both	11
	6b	Any changes to trial outcomes after the trial commenced, with reasons		11
**Sample size**	7a	How sample size was determined	Method of calculation, number of clusters(s) (and whether equal or unequal cluster sizes are assumed), cluster size, a coefficient of intracluster correlation (ICC or *k*), and an indication of its uncertainty	12-13
	7b	When applicable, explanation of any interim analyses and stopping guidelines		12-13
**Randomisation:**				
**Sequence generation**	8a	Method used to generate the random allocation sequence		12
	8b	Type of randomisation; details of any restriction (such as blocking and block size)	Details of stratification or matching if used	-
**Allocation concealment mechanism**	9	Mechanism used to implement the random allocation sequence (such as sequentially numbered containers), describing any steps taken to conceal the sequence until interventions were assigned	Specification that allocation was based on clusters rather than individuals and whether allocation concealment (if any) was at the cluster level, the individual participant level or both	11
**Implementation**	10	Who generated the random allocation sequence, who enrolled participants, and who assigned participants to interventions	Replace by 10a, 10b and 10c	
	10a		Who generated the random allocation sequence, who enrolled clusters, and who assigned clusters to interventions	8-9, 12
	10b		Mechanism by which individual participants were included in clusters for the purposes of the trial (such as complete enumeration, random sampling)	8-9
	10c		From whom consent was sought (representatives of the cluster, or individual	Suppl. Appendix Section 11

			cluster members, or both), and whether consent was sought before or after randomisation	
**Blinding**	11a	If done, who was blinded after assignment to interventions (for example, participants, care providers, those assessing outcomes) and how		-
	11b	If relevant, description of the similarity of interventions		10-11
**Statistical methods**	12a	Statistical methods used to compare groups for primary and secondary outcomes	How clustering was taken into account	12-13
	12b	Methods for additional analyses, such as subgroup analyses and adjusted analyses		12-13
**Results**				
**Participant flow (a diagram is strongly recommended)**	13a	For each group, the numbers of participants who were randomly assigned, received intended treatment, and were analysed for the primary outcome	For each group, the numbers of clusters that were randomly assigned, received intended treatment, and were analysed for the primary outcome	13
	13b	For each group, losses and exclusions after randomisation, together with reasons	For each group, losses and exclusions for both clusters and individual cluster members	13-14
**Recruitment**	14a	Dates defining the periods of recruitment and follow-up		13 and Suppl. Appendix Section 1
	14b	Why the trial ended or was stopped		-
**Baseline data**	15	A table showing baseline demographic and clinical characteristics for each group	Baseline characteristics for the individual and cluster levels as applicable for each group	Table 3
**Numbers analysed**	16	For each group, number of participants (denominator) included in each analysis and whether the analysis was by original assigned groups	For each group, number of clusters included in each analysis	13-14
**Outcomes and estimation**	17a	For each primary and secondary outcome, results for each group, and the estimated effect size and its precision (such as 95% confidence interval)	Results at the individual or cluster level as applicable and a coefficient of intracluster correlation (ICC or k) for each primary outcome	Table 4
	17b	For binary outcomes, presentation of both absolute and relative effect sizes is recommended		Table 4
**Ancillary analyses**	18	Results of any other analyses performed, including subgroup analyses and adjusted analyses, distinguishing pre-specified from exploratory		Table 4 and Suppl. Appendix Sections 9 and 10
**Harms**	19	All important harms or unintended effects in each group (for specific guidance see CONSORT for harms[i])		-
**Discussion**				
**Limitations**	20	Trial limitations, addressing sources of potential bias, imprecision, and, if relevant, multiplicity of analyses		17
**Generalisability**	21	Generalisability (external validity, applicability) of the trial findings	Generalisability to clusters and/or individual participants (as relevant)	18
**Interpretation**	22	Interpretation consistent with results, balancing benefits and harms, and considering other relevant evidence		18
**Other information**				
**Registration**	23	Registration number and name of trial registry		6
**Protocol**	24	Where the full trial protocol can be accessed, if available		7-8 and reference 14
**Funding**	25	Sources of funding and other support (such as supply of drugs), role of funders		6

13. Data collection instrument: ANC logbook

Fig S5: Page 1 (left page) of the Mozambique national ANC logbook (each row represent one ANC visit).

Livro de Registos da Consulta Pré-Natal - MOD SIS-B01 **Ano ________________**

	Nº de ordem mensal	Data da CPN (D/M)	Nome da utente	Idade	Tipo de CPN (marque com X o que se aplicar): Primeira	Seguinte	Quarta CPN	IG em semanas	Tensão arterial ≥ 140/90 mmHg: Sim	Não	Desnutrição – IMC (para 1ª CPN): < 16 kg/m2 (grave)	16-18.5 kg/m 2 (moderada)	PB entre 19-22 cm na CPN seguintes	Hemoglobina <8mg/dl: Sim	Não	Proteinuria: Sim	Não	Despiste e tratamento de sífilis e outras ITS: Úlcera genital (marque X)	Leucorreia (marque X)	Teste de sífilis (marque com circulo na data do diagnóstico) - Postivo(P); Negativo(N), Não Fez (Nf)	Tratamento da sífilis na grávida (marque com X apenas uma opção): 1ª dose	2ª dose	3ª dose	Parceiro: Teste de sífilis (marque com circulo na data do diagnóstico) - Postivo(P); Negativo(N), Não Fez (Nf)	Parceiro: Tratamento da sífilis (marque com X apenas uma opção): 1ª dose	2ª dose	3ª dose	Exame da mama (presen-ça de nódulos)	VAT (marque com X apenas um): 1ª dose	2ª dose	Dose de reforço	Prevenção da anemia: recebe sal ferroso c/ ácido fólico	Recebe desparasitante	Seroestado HIV na 1ª CPN (marque com X apenas um): Desconhecido	Negativo	Positivo	Se positivo, em TARV?
	1	2	3	4	5	6	7	8	9	10	11	12	13	14	15	16	17	18	19	20	21	22	23	24	25	26	27	28	29	30	31	32	33	34	35	36	37
1																				P N Nf				P N Nf				P N									
2																				P N Nf				P N Nf				P N									
3																				P N Nf				P N Nf				P N									
4																				P N Nf				P N Nf				P N									
5																				P N Nf				P N Nf				P N									
6																				P N Nf				P N Nf				P N									
7																				P N Nf				P N Nf				P N									
8																				P N Nf				P N Nf				P N									
9																				P N Nf				P N Nf				P N									
10																				P N Nf				P N Nf				P N									
11																				P N Nf				P N Nf				P N									
12																				P N Nf				P N Nf				P N									
13																				P N Nf				P N Nf				P N									
14																				P N Nf				P N Nf				P N									
15																				P N Nf				P N Nf				P N									
16																				P N Nf				P N Nf				P N									
17																				P N Nf				P N Nf				P N									
18																				P N Nf				P N Nf				P N									
19																				P N Nf				P N Nf				P N									
20																				P N Nf				P N Nf				P N									
21																				P N Nf				P N Nf				P N									
22																				P N Nf				P N Nf				P N									
23																				P N Nf				P N Nf				P N									
24																				P N Nf				P N Nf				P N									
25																				P N Nf				P N Nf				P N									
																				Testadas / P				Testados= / P=				Total= / P=									

Fig S6: Page 2 of the Mozambique national ANC logbook (each row represent one ANC visit).

Livro de Registos da Consulta Pré-Natal - MOD SIS-B01 **Ano** ________________

	Prevenção da transmissão vertical do HIV (marque com X o que se aplica)													Prevenção da malária									
	Resultado do **Teste HIV** na CPN			Profilaxia com CTZ				Recebe **profilaxia ARVs** durante o mês						Tratamento Intermitente Preventivo da Malária (TIP)			Recebe REMTIL						
								**Inicia toma de**															
								**Biprofilaxia**															
	Resultado do **1º teste**	Resultado do **teste seguinte**	Código de PTV	Iniciou CTZ no mês	Continua toma de CTZ no mês	CD4<350 ou estadio OMS III ou IV	Iniciou TARV no mês	**AZT** (≥14s)	**NVP** (≥28s)	**Monoprofilixia** (NVP)	Outro regime (qual?)	**Continua** (qual?)	Resultado do teste HIV do parceiro na CPN	1ª Dose	2ª Dose	3ª Dose	Na 1ª CPN	Na CPN seguinte	Parceiro presente na consulta	Diagnóstico e Tratamento	**Referência para outra US ou Serviço**	**Observações**	**Nome do provedor de cuidados**
	38	39	40	41	42	43	44	45	46	47	48	49	50	51	52	53	54	55	56	57	58	59	60
1	P N I NF	P N I NF											P N I NF										
2	P N I NF	P N I NF											P N I NF										
3	P N I NF	P N I NF											P N I NF										
4	P N I NF	P N I NF											P N I NF										
5	P N I NF	P N I NF											P N I NF										
6	P N I NF	P N I NF											P N I NF										
7	P N I NF	P N I NF											P N I NF										
8	P N I NF	P N I NF											P N I NF										
9	P N I NF	P N I NF											P N I NF										
10	P N I NF	P N I NF											P N I NF										
11	P N I NF	P N I NF											P N I NF										
12	P N I NF	P N I NF											P N I NF										
13	P N I NF	P N I NF											P N I NF										
14	P N I NF	P N I NF											P N I NF										
15	P N I NF	P N I NF											P N I NF										
16	P N I NF	P N I NF											P N I NF										
17	P N I NF	P N I NF											P N I NF										
18	P N I NF	P N I NF											P N I NF										
19	P N I NF	P N I NF											P N I NF										
20	P N I NF	P N I NF											P N I NF										
21	P N I NF	P N I NF											P N I NF										
22	P N I NF	P N I NF											P N I NF										
23	P N I NF	P N I NF											P N I NF										
24	P N I NF	P N I NF											P N I NF										
25	P N I NF	P N I NF											P N I NF										
	Testadas / P / N	Testadas / P / N											Testados / P										
